# Population genetic structure in a self‐compatible hermaphroditic snail is driven by drift independently of its contemporary mating system

**DOI:** 10.1002/ece3.70162

**Published:** 2024-08-13

**Authors:** Cansu Çetin, Jukka Jokela, Philine G. D. Feulner, Tamara Schlegel, Nadine Tardent, Otto Seppälä

**Affiliations:** ^1^ Department of Aquatic Ecology Swiss Federal Institute of Aquatic Science and Technology Dübendorf Switzerland; ^2^ Institute of Integrative Biology ETH Zurich Zurich Switzerland; ^3^ Institute of Environmental Sciences, Faculty of Biology Jagiellonian University Kraków Poland; ^4^ Department of Fish Ecology and Evolution, Centre of Ecology, Evolution and Biogeochemistry EAWAG Swiss Federal Institute of Aquatic Science and Technology Kastanienbaum Switzerland; ^5^ Division of Aquatic Ecology and Evolution, Institute of Ecology and Evolution University of Bern Bern Switzerland; ^6^ Research Department for Limnology Universität Innsbruck Mondsee Austria

**Keywords:** genetic structure, inbreeding, RAD sequencing, relatedness, small populations

## Abstract

Genetic drift, gene flow, and natural selection commonly influence population genetic diversity. In populations of self‐compatible hermaphrodites, the mating system (e.g., self‐fertilization) further reduces individual heterozygosity. Furthermore, selfing, as a form of inbreeding, significantly impacts genetic drift by reducing effective population size (*N*_e_). This can potentially accelerate genetic drift, particularly in small populations where self‐fertilization is likely during founder events. To investigate the roles of genetic drift and contemporary mating system in populations of the freshwater snail *Lymnaea stagnalis*, we examined their effective population sizes (*N*_e_) and Tajima's *D* values, which reflect genetic drift over extended time periods, as well as estimates of within‐population selfing rates and pairwise relatedness reflecting contemporary mating system. We used 4054 SNP markers obtained using restriction site associated DNA (RAD) sequencing from individuals in five snail populations originating from geographically closely located ponds. We found strong population genetic structure and differences in genetic diversity among populations. Covariation between genetic diversity and *N*_e_ estimates and Tajima's *D* values suggested drift being an important determinant of genetic diversity and structure in these populations. However, this effect was independent of the contemporary mating system, as indicated by the similarity of selfing rates and relatedness estimates among populations. Thus, founder events (possibly including historical inbreeding) and/or drift due to small sizes of *L. stagnalis* populations are likely to explain their genetic structure and limit within‐population genetic diversity.

## Graphical abstract

We investigated the roles of genetic drift and contemporary mating system in shaping the genetic diversity of freshwater snail populations by analyzing effective population sizes, Tajima's *D* values, selfing rates, and relatedness using 4054 SNP markers from RAD sequencing. Our findings revealed strong genetic structure and diversity differences among populations, with genetic drift being a significant factor independent of the contemporary mating system. This suggests that founder events and small population sizes contribute to the genetic structure and limited within‐population diversity in *Lymnaea stagnalis*.

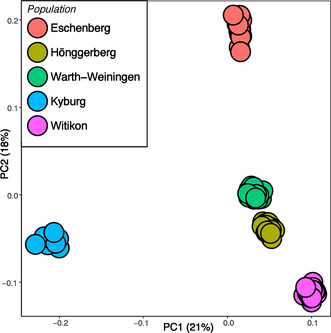

## 1 INTRODUCTION

Genetically diverse populations are assumed to have the highest evolutionary potential and, thus, the lowest probability of extinction in changing environments (Hoffmann et al., 2017). Directional natural selection reduces genetic diversity owing to the fixation of alleles with positive fitness effects (Charlesworth et al., 1993; Charlesworth & Charlesworth, 2010; Hohenlohe et al., 2010), while genetic drift reduces variation through the random loss of rare gene variants (Byrne & Nichols, 1999; Wright, 1931). The contemporary mating system and inbreeding history, including biparental inbreeding and self‐fertilization, can further impact genetic diversity by reducing individual heterozygosity (Crnokrak & Roff, 1999; DeRose & Roff, 1999) and increasing levels of pairwise relatedness (Ingvarsson, 1998; Wang, 2007, 2014). Additionally, inbreeding creates identity disequilibrium (i.e., correlations of heterozygosity), increasing genetic differences among individuals, and may reduce individual fitness due to inbreeding depression (Coutellec & Lagadic, 2006; Henry et al., 2003; Noël et al., 2017).

The interplay of genetic drift and the mating system is especially important for populations experiencing extinction‐recolonization dynamics (Ellegren & Galtier, 2016). This is because stochastic demographic processes referring to the random changes in the survival and reproductive rates of individuals can enhance the impact of genetic drift when the effective population size (*N*_e_) is small (Pannell & Charlesworth, 2000). Such effects could be even stronger in species that can self‐fertilize because the number of founders could be as low as one, making populations extremely prone to increased homozygosity during (re)colonization events (Jarne, 1995). While past inbreeding events may have shaped genetic diversity (together with the effect of small population size), understanding how the contemporary mating system contributes to genetic variation in such populations remains elusive (but see Diaz‐Martin et al., 2023; Ingvarsson, 1998). However, addressing this gap is essential for our ability to decipher the adaptive potential and extinction risks of organisms with small and/or fluctuating populations because the contemporary mating system directly influences, for example, individual heterozygosity and relatedness (Byrne & Nichols, 1999; Keller & Waller, 2002; Wright, 1931).

Modern genome‐wide multilocus analyses allow for the estimation of different population genetic processes, such as genetic drift (reflected by *N*_e_ and linkage disequilibrium) and details of the mating system (e.g., relatedness estimates based on identity by descent (IBD) and selfing rate) in nature (Kardos et al., 2015; Puurtinen et al., 2004). We examined the roles of genetic drift and contemporary mating system in determining the genetic diversity and structure in natural populations of the self‐compatible hermaphroditic freshwater snail, *Lymnaea stagnalis*. In this system, both processes (i.e., drift, mating system) could have large roles in determining genetic diversity. This is because, first, *L. stagnalis* commonly inhabits small ponds where extinctions and founder events can accentuate genetic drift. In fact, genetic diversity in spatially closely located *L. stagnalis* populations varies considerably, and genetic differentiation among them is often high (Kopp et al., 2012; Puurtinen et al., 2004). However, earlier population genetic analyses of *L. stagnalis* used co‐dominant markers covering fewer loci (e.g., microsatellites, allozyme polymorphism) (Coutellec‐Vreto et al., 1994; Kopp et al., 2012; Puurtinen et al., 2004; Ritland & Jain, 1984), which provided less precise estimates for genetic diversity, linkage, and relatedness. Second, although preferring outcrossing (Cain, 1956), *L. stagnalis* can commit to the most extreme form of inbreeding, self‐fertilization, which can help them while founding populations (Kopp et al., 2012). While selfing can be advantageous for colonizing new environments, it can also lead to reduced genetic diversity within populations. Additionally, population bottlenecks and founder events may have purged recessive deleterious mutations, which may be the reason why most *L. stagnalis* populations do not show strong inbreeding depression (Coutellec & Lagadic, 2006; Koene et al., 2008; Puurtinen et al., 2007). Therefore, our study system allows for testing the role of the contemporary mating system on genetic diversity without strong selection against selfing influencing the results.

Here, we examined both population‐ and individual‐level genetic parameters in five *L. stagnalis* populations using 4054 SNP markers. SNP markers are powerful for estimating the linkage of alleles, selfing rates, *N*_e_, Tajima's *D* (informing about deviations in rare allele frequencies from neutral expectations, which can indicate demographic events such as bottlenecks), and relatedness between individuals (Attard et al., 2018; Lemopoulos et al., 2019). Variation in the estimates reflecting genetic drift (e.g., *N*_e_ and Tajima's *D*) and their covariation with genetic variation would suggest the effect of genetic drift (e.g., founder events including historical inbreeding due to selfing/biparental inbreeding) being an important determinant of genetic diversity. Furthermore, variation in mating system estimates (relatedness and selfing rates) and their covariation with genetic diversity would imply that the contemporary mating system plays a crucial role in shaping genetic diversity. Thus, we predicted that populations with more genetic drift (i.e., lower *N*_e_ and higher deficit of rare alleles indicated by Tajima's *D*) and/or with more contemporary inbreeding (high selfing rates and/or relatedness) would have the lowest genetic diversity.

## 2 MATERIALS AND METHODS

### 2.1 Sampling, DNA extraction, and sequencing

**FIGURE 1:**
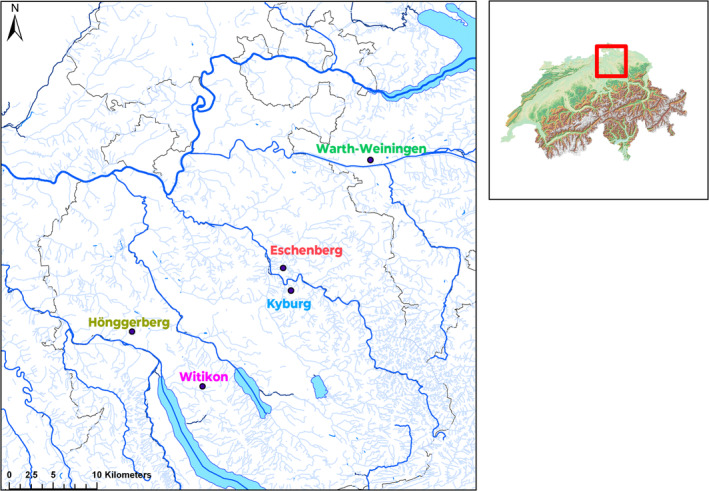
Locations of the investigated *Lymnaea stagnalis* populations in northern Switzerland. Data were provided by Geodata CH (geo.admin.ch), and the map was produced using ArcGis Map (version 10.7.1, ESRI).

We collected a total of 92 *L. stagnalis* snails from five ponds near Zürich in northern Switzerland in 2017 (see Figure 1 and Table 1). Pairwise distances between the populations ranged from 2.5 to 34 km. Because larger ponds can contain larger populations and provide more migrants, we calculated the area of each pond to the nearest m^2^ using a topographic map (map.geo.admin.ch). Furthermore, because landscape may influence the isolation of the populations, we also considered the elevation of the studied ponds.

**TABLE 1:** Location information and genetic diversity values for the examined *L. stagnalis* populations based on the non‐thinned dataset.

Population	Eschenberg	Hönggerberg	Warth‐Weiningen	Kyburg	Witikon
Code	EB	HB	WW	KB	WT
Coordinates	47°28′29.9″ N, 8°43′46.2″ E	47°24′42.2″ N, 8°29′57.9″ E	47°35′01.4″ N, 8°51′51.6″ E	47°27′04.8″ N, 8°44′26.2″ E	47°21′15.1″ N, 8°36′16.8″ E
*N*	19	19	19	17	18
Elevation (m)	527	516.7	423	624	613
Area (m^2^)	429	218	307	NA	308
Polymorphic sites	2010	2685	2506	1454	1655
Private alleles	219	200	177	229	90
*H*_e_	0.18	0.21	0.21	0.13	0.16
*H*_o_	0.14	0.15	0.15	0.09	0.13
*π*	0.0033	0.0038	0.0038	0.0024	0.0029

*Note*: *N* refers to the number of individuals sampled from each population. Each pond's elevation (m) and area (m^2^) are indicated. Genetic diversity for each population, including the number of polymorphic sites, private alleles, expected heterozygosity (*H*_e_), observed heterozygosity (*H*_o_), and nucleotide diversity (*π*) is displayed.

We extracted genomic DNA from the head tissue of each collected snail using the CTAB method (modified from Doyle and Doyle (1987)) followed by magnetic bead cleanup (see Text S1). We measured DNA concentrations and quality using Qubit 2.0 Fluorometer (dsDNA, HS, Invitrogen, Carlsbad, California, USA) and ND1000 spectrophotometer (Thermo Fisher, Waltham, Massachusetts, USA), respectively. We stored DNA in RNase/DNase‐free water (Sigma‐Aldrich, MO) at −20°C until library preparation. RAD library preparation and single digest RAD‐sequencing (restriction enzyme SbfI) using a 100 ‐bp single‐end Illumina HiSeq2000 platform were conducted by Floragenex (Oregon, USA) using the protocol of Baird et al. (2008). Briefly, sequencing adapters and multiplex sample indices (barcodes) were ligated to SbfI digested gDNA, and fragments were selected for a size of 200–400 bp before sequencing. To segregate sequences by individual barcodes (i.e., sample indices), we demultiplexed the FASTQ sequence file and simultaneously trimmed all sequences to 85 bp using *process_radtags* module of STACKS v. 1.40 (Catchen et al., 2013).

### 2.2 Genotyping and filtering

We mapped the 85 bp sequenced reads to the *L. stagnalis* reference genome (GCA_900036025.1) using Bowtie2 v. 2/2.2.3 (Langmead & Salzberg, 2012), followed by genotype calling using FreeBayes v. 1.0.0 (Garrison & Marth, 2012). We used the dDocent pipeline (Puritz et al., 2014) with minor changes to filter variant calls from the raw vcf file (Table S1). We applied a minimum mean depth filter of 10 and a maximum mean depth filter of 550 using vcftools v.0.1.16 (Danecek et al., 2011). We removed indels, kept only biallelic sites, and restricted the data to variants called in 80% of individuals across the populations. We excluded 17 RAD loci (85 bp sequenced reads) containing more than five SNPs and removed sites that were present less than five times in the overall dataset to reduce erroneous calls. This resulted in a vcf file with 4054 SNPs across populations (average mean SNP depth: 252.8; average mean individual depth: 252.5). This “non‐thinned dataset” kept as many loci as possible and was used to analyze the genetic diversity of the populations (heterozygosity, nucleotide diversity, number of private alleles). We created another dataset in which we randomly kept one SNP per RAD locus to avoid the inclusion of physically linked markers in analyses of population structure and relatedness that could be biased from this (see Table S1 for detailed filtering steps). This “thinned dataset” consisted of 2263 SNPs across populations and was used to analyze population structure (*F*_ST_, PCA), genetic drift estimates (linkage disequilibrium and Tajima's *D*), and mating system estimates (inbreeding, relatedness, and selfing rates).

### 2.3 Population genetic diversity and differentiation

We examined genetic diversity in the studied snail populations using the nonthinned dataset. We calculated nucleotide diversity (*π*, the average number of pairwise nucleotide differences between sequences in a sample (Nei & Li, 1979)) for each population using vcftools and corrected for the total number of sequenced nucleotides. We then calculated the number of polymorphic sites, the number of private alleles, expected heterozygosity (*H*_e_), and observed heterozygosity (*H*_o_) using the “populations” module of the STACKS software v.2.41 (Catchen et al., 2013; Rochette et al., 2019). Then, we examined genetic differentiation among populations using the thinned dataset. We conducted a principal components analysis (PCA) using snprelate (Zheng et al., 2012). We also calculated fixation index *F*_ST_ using pairwise AMOVA directly from the output of the population script of the STACKS v.2.41. (Weir, 1996). A locus was included in the pairwise *F*_ST_ analyses only if it was present in at least 85% of individuals within a population and in at least 50% of individuals across populations. This filtering resulted in the exclusion of three of the 2263 loci. We then calculated *F*_ST_ values using the “populations” module of the STACKS software v.2.41.

### 2.4 Estimates of genetic drift and historical inbreeding

To approximate the rate of genetic drift and potential historical inbreeding due to founder events, we first estimated effective population size (*N*_e_) using the nonthinned dataset with the LDN_e_ (linkage disequilibrium) method (Waples & Do, 2008) in NeEstimator v2.1 (Do et al., 2014). We analyzed each population separately, keeping only polymorphic loci for each population. We estimated confidence intervals for *N*_e_ values using a parametric approach implemented in the program. We used a “two allele” minimum for each locus within each population based on recommendations from Waples and Do (2010) for sample sizes smaller than 25. We also tested the effect of minor allele filtering (MAF) on LDN_e_ estimates using the settings in NeEstimator v2.1. We tested MAFs of 0.05 and no filter (0) per population. The *N*_e_ estimates did not change between the filters. The 95% CI was slightly larger for the 0.05 filter compared to no filter (0). We thus report the results of no additional MAF per population. Second, using the non‐thinned dataset, we compared the observed number of segregating sites and nucleotide diversity (*π*) using Tajima's *D* test (Tajima, 1989), which informs about deviations from neutral expectations. Positive values indicate a deficiency of rare alleles, while negative values suggest an excess of rare alleles relative to neutral expectations. We calculated Tajima's *D* in 10‐kb sliding windows using VCFtools v. 0.1.16 (Danecek et al., 2011) and reported the genome‐wide average values across windows for each population. We aimed for a window size that is small enough to minimize recombination in the region but large enough to contain enough data. Additionally, we performed Tajima's *D* test on the dataset with no MAF applied to assess if minor allele filtering significantly impacted Tajima's *D* estimates. Lastly, to test the null hypothesis of linkage equilibrium of SNP loci, we estimated the index of association, *I*_A_ (Brown et al., 1980), that measures the pairwise allele correlations in the population. We also calculated the standard index of association (*r*_d_) and corresponding *p*‐values for *I*_A_ and *r*_d_ using 1000 permutations with the “poppr” package (Kamvar et al., 2014).

### 2.5 Mating system estimates

To evaluate the importance of the contemporary mating system on the patterns of genetic variation, we estimated rates of inbreeding, relatedness, and self‐fertilization in each population using the thinned dataset. We first estimated the inbreeding rate in five populations using the fixation index *F*_IS_, which is the proportional reduction in heterozygosity due to inbreeding. We calculated the *F*_IS_ using the “populations” module of the STACKS software v.2.41.

We estimated selfing rates, *s*, from *F*_IS_ using the formula *s = 2 F*_IS_/(1 + *F*_IS_) (Wright, 1969). We also estimated selfing rates, *s*(*g*_2_), using a maximum‐likelihood approach based on the identity disequilibrium (*g*_*2*_) with the software Robust Multilocus Estimation of Selfing (RMES) (David et al., 2007). RMES uses the distribution of multilocus heterozygosity, which is considered more reliable compared to estimates derived from *F*_IS_ (Bürkli et al., 2017; David et al., 2007). Because RMES cannot calculate *s*(*g*_*2*_) using more than 1000 SNPs, we took five subsamples of 1000 SNPs from the data for each population and calculated *s*(*g*_*2*_) for each of them. We report the average *s*(*g*_*2*_) of each population. We used 1000 iterations of RMES to generate *p*‐values for *s*(*g*_*2*_) (Miller et al., 2014).

To evaluate the rates of bi‐ and uniparental inbreeding, we estimated the relatedness among all pairwise genotypes and calculated individual inbreeding coefficients, *f*. Relatedness is defined as the probability of identity by descent of two alleles at a given locus that two individuals share (Keller & Waller, 2002; Wright, 1922). We estimated relatedness using the triadic likelihood estimator (TrioML) of Wang (2007) with the R package “related” v.1 (Pew et al., 2015). This estimator is considered more robust toward inbreeding and bias due to the small sample size and genotyping errors compared to the five commonly used moment estimators and the dyadic likelihood method (Wang, 2007). We used the settings “unknown allele frequencies” (i.e., allele frequencies calculated directly from the genetic data) and “inbreeding.” We also tested whether these estimates differed among populations. We used individual TrioML inbreeding and pairwise TrioML relatedness estimates as response variables and population as a factor in a linear model in SPSS 26.

### 2.6 Relationships between genetic diversity, genetic drift, and mating system estimates

We estimated the determinants of genetic diversity in the examined populations by calculating Pearson correlations among nucleotide diversity (*π*) and environmental characteristics of ponds (elevation and area of the ponds) and the most relevant measures for drift (*N*_e_ and Tajima's *D*) and mating type (selfing rates) using SPSS v. 26. Because relatedness (*r*) and *F*_IS_ can be influenced by other factors such as biparental inbreeding and population size, we did not use them in the correlations with genetic diversity (Bierne et al., 2000; Szulkin et al., 2010). Additionally, we examined possible correlations between the explanatory variables before examining their correlations with genetic diversity. The variables representing drift and mating type did not covary (Table S2).

## 3 RESULTS

### 3.1 Population genetic diversity and differentiation

Populations differed in their observed heterozygosity (range: 0.09–0.15), the number of polymorphic sites (range: 1454–2685 out of 4054 SNPs), nucleotide diversity (*π*) (range: 0.0024–0–0038), and the number of private alleles (range: 90–229) (Table 1). All populations had lower observed than expected heterozygosity (Table 1). Populations of Hönggerberg and Warth‐Weiningen were the most genetically diverse, having almost twice as many polymorphic sites as the least diverse population, Kyburg. Although Kyburg and Witikon were the least diverse populations, the number of private alleles in them differed more than twofold from each other (Table 1). The elevation and area of the ponds did not covary with their genetic diversity (Table 1, *r* = −.86; *p* = .14 and *r* = −.44; *p* = .56, respectively).

**FIGURE 2:**
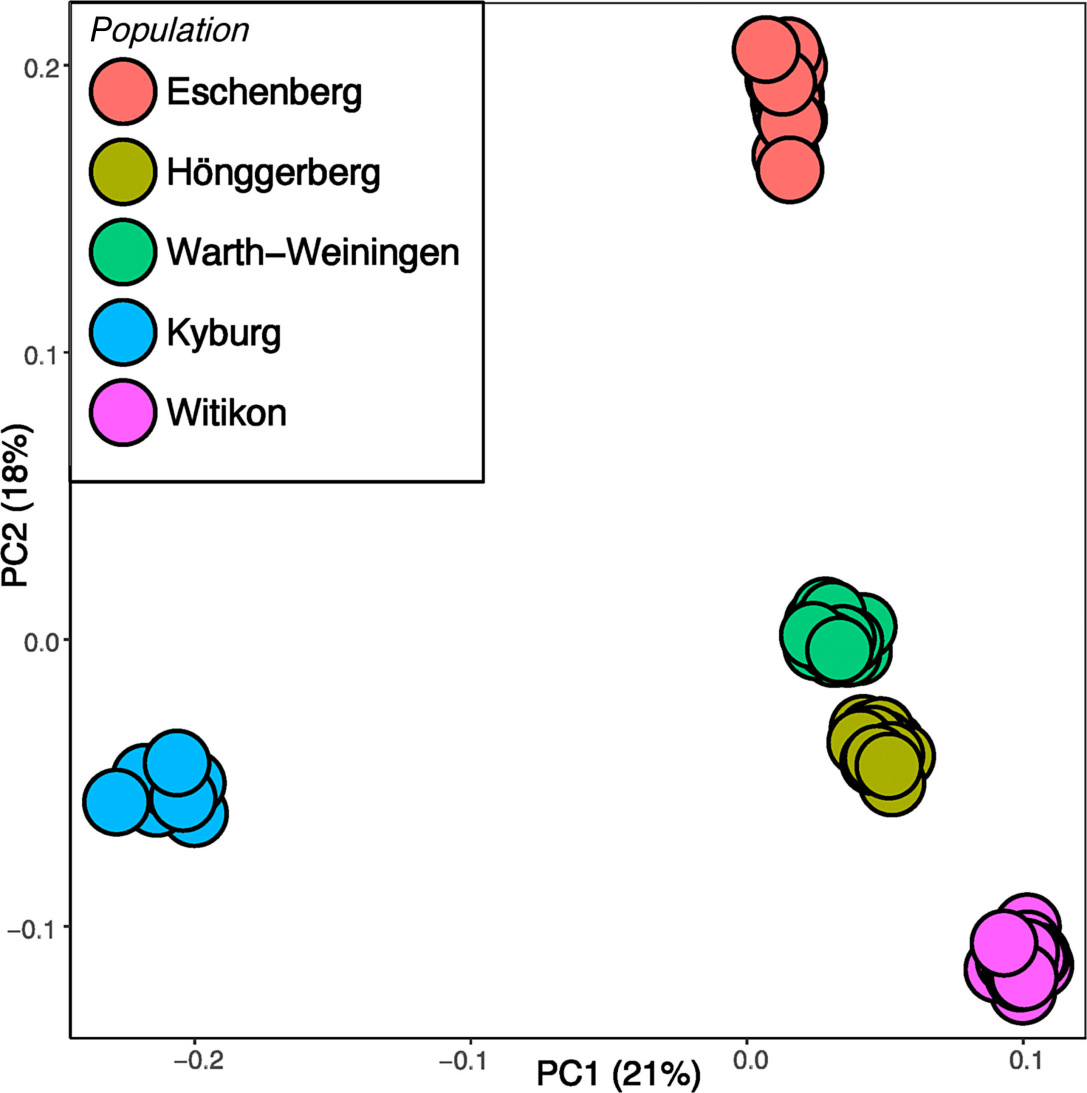
A scatterplot showing individual variation in principal component (PC) scores derived from principal component analysis (PCA) of the genomic data (2263 SNPs). The amounts of variation explained by each PC axis are given as percentages.

Examined populations were clearly differentiated from each other based on pairwise *F*_ST_ estimates (overall *F*_ST_ = 0.17, Table 2). PCA resolved five distinct and nonoverlapping clusters separating the populations, the first two principal components explaining 21% and 18% of the total variation (Figure 2). The genetic differentiation of populations showed no obvious spatial pattern; even the closest populations, Kyburg and Eschenberg, exhibited large genetic differences in allele frequencies (pairwise *F*_ST_ = 0.28, Table 2, Figure 2). The most genetically diverse populations, Hönggerberg and Warth‐Weiningen, were genetically most similar (pairwise *F*_ST_ = 0.14, Table 2, Figure 2). In contrast, the genetic differentiation between the two least diverse populations, Kyburg and Witikon, was the highest (pairwise *F*_ST_ = 0.32, Table 2, Figure 2).

**TABLE 2:** Pairwise *F*_ST_ estimates between all five populations. The degree of color intensity, as indicated by the scale bar, corresponds to increasing *F*_ST_ values.

Sites	Eschenberg	Hönggerberg	Warth‐Weiningen	Kyburg
Hönggerberg	0.19			
Warth‐Weiningen	0.19	0.14		
Kyburg	0.28	0.23	0.24	
Witikon	0.26	0.18	0.21	0.32
0.14 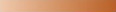 0.32				

### 3.2 Estimates of genetic drift and historical inbreeding

Effective population size (*N*_e_) estimates of the examined populations were significantly different from each other, ranging from 40 to 231 (Table 3). The highest *N*_e_ estimates were obtained for Warth‐Weiningen and Hönggerberg, while the lowest was for Witikon (Table 3). The effective population sizes of Kyburg and Eschenberg were intermediate (Table 3). All populations had a positive Tajima's *D* value, even without applying any MAF (Table 3, Table S3). For all populations except Warth‐Weiningen, linkage disequilibrium (*r*_d_) differed significantly from zero (Table 3). Genetically most diverse populations, Warth‐Weiningen and Hönggerberg, had lower *r*_d_ compared to other populations (Table 3). Populations' *N*_e_ estimates and Tajima's *D* values covaried with their genetic diversity (*r* = .98; *p* = .02 and *r* = −.95; *p* = .05, respectively, Table S2).

**TABLE 3:** Estimates for genetic drift based on the thinned dataset.

Population	*N*_e_ (95% CI)	Tajima's *D*	*I*_A_ (*p* value)	*r*_d_ (*p* value)
Eschenberg	91 (65–149)	0.99	**3.37 (.001)**	**0.0031 (.001)**
Hönggerberg	195 (97–4763)	0.63	**2.06 (.001)**	**0.0015 (.001)**
Warth‐Weiningen	231 (105–infinite)	0.81	1.94 (.098)	0.0015 (.081)
Kyburg	90 (57–195)	0.96	**1.69 (.002)**	**0.0021 (.002)**
Witikon	41 (29–65)	1.19	**5.66 (.001)**	**0.0064 (.001)**

*Note*: Effective population sizes (*N*_e_) and their associated 95% confidence intervals (CI), Tajima's *D*, and estimates of linkage disequilibrium (the index of association, *I*_A_, based on pairwise allele correlations, and the index of association standardized for the number of loci, *r*_d_) are shown. Significant estimates (*p* < .05) for *I*_A_, and *r*_d_ are shown in bold.

### 3.3 Mating system estimates

The two most diverse populations, Hönggerberg and Warth‐Weiningen, also had the highest population level inbreeding estimates (*F*_IS_) (Table 4). In comparison, the two least diverse populations, Kyburg and Witikon, had the lowest *F*_IS_ values (Table 4). Selfing rate estimates based on *F*_IS_ values, *s*(*F*_IS_), ranged between 0.18 and 0.31 (Table 4). However, selfing rate estimates based on identity disequilibrium, *s*(*g*_*2*_), are more suited for multilocus data (*F*_IS_ averages across the markers and could be inflated by linkage disequilibrium caused by other factors than selfing (Bürkli et al., 2017; David et al., 2007)). The obtained *s*(*g*_*2*_) estimates were lower than *s*(*F*_IS_) values but significantly higher than zero for all populations except Hönggerberg (Table 4), ranging between 0.04 and 0.10 (Table 4). There was no significant correlation between selfing rates and population genetic diversity (*r* = −.72; *p* = .28, Table S2) nor among variables representing drift and nonrandom mating (Table S2).

**TABLE 4:** Estimates for nonrandom mating based on the thinned dataset.

Population	*F*_IS_	*s*(*F*_IS_)	*g*_*2*_	*s*(*g*_*2*_)	*f*	*r*
Eschenberg	0.14	0.25	0.02	**0.07**	0.25	.05
Hönggerberg	0.17	0.29	0.01	0.04	0.26	.04
Warth‐Weiningen	0.18	0.31	0.02	**0.08**	0.28	.04
Kyburg	0.11	0.20	0.02	**0.08**	0.28	.04
Witikon	0.10	0.18	0.03	**0.10**	0.23	.05

*Note*: The table presents the inbreeding coefficient (*F*_IS_) and selfing rate for each population, derived from *s*(*F*_IS_). It also shows the selfing rate, *s*, which is calculated using the identity disequilibrium (*g*_*2*_, which is used to estimate *s*(*g*_*2*_)). Mean inbreeding (*f*) and relatedness (*r*) are also provided for each population, calculated using within‐population allele frequencies. Significant estimates (*p* < .05) for *s*(*g*_*2*_) are shown in bold.

**FIGURE 3:**
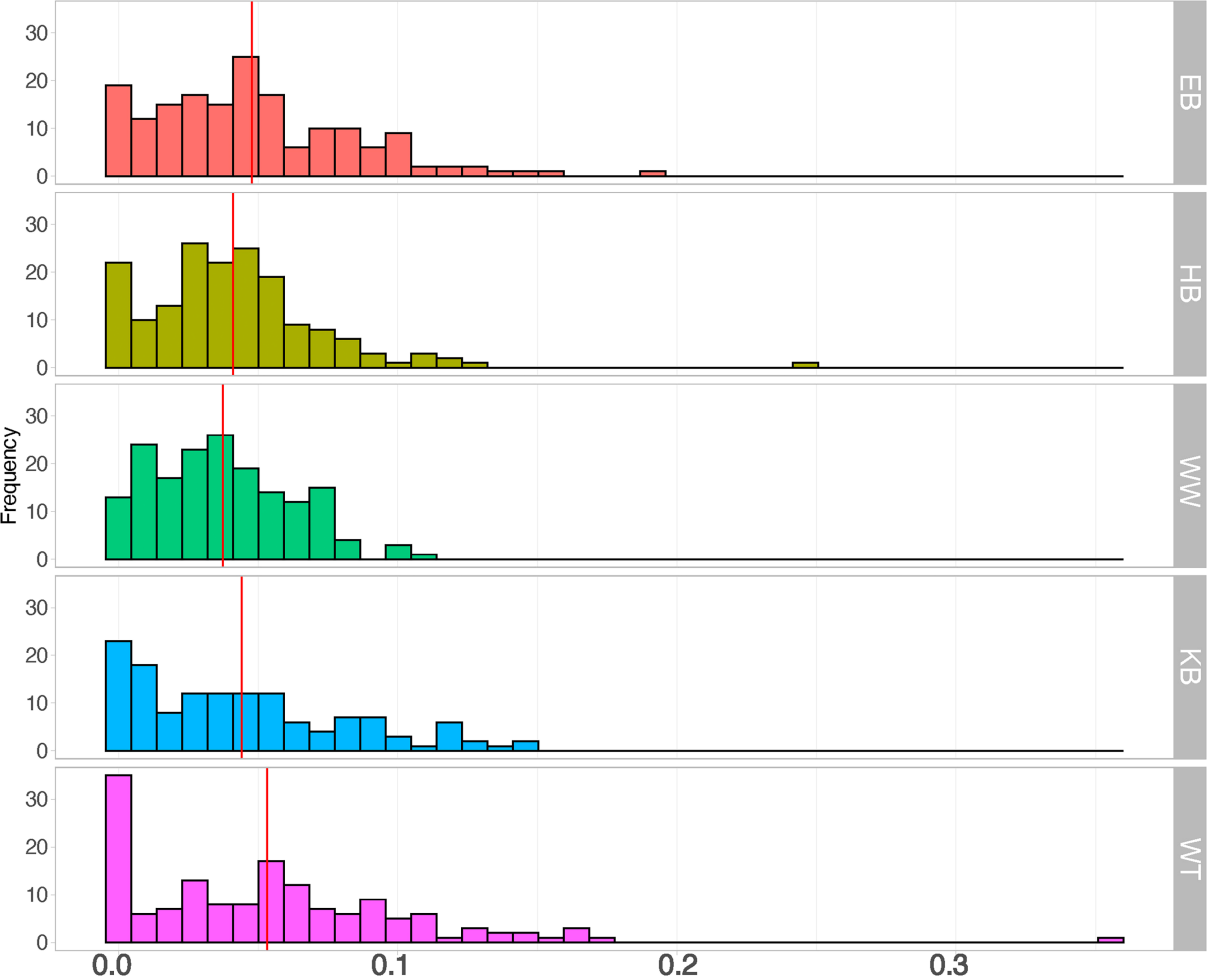
Pairwise relatedness estimates from the probability‐based estimator TrioML (Wang, 2007) performed on each of the five populations separately, using within‐population allele frequencies in corresponding populations. The red lines indicate the mean values for each population.

Using polymorphic sites in each population as a reference, mean relatedness values, *r*, were low, ranging from 0.04 to 0.05 (Table 4), but still significantly different among populations (*p* < .01). More than half (55%) of all pairwise *r* values within the populations exhibited a level of relatedness characteristic of first cousins (0.125) to second cousins (0.03125) (Figure 3). Mean individual level inbreeding coefficients, *f*, of populations were high overall (mean value of 0.26) and did not significantly differ among them (*p* = .26). The distributions of relatedness and inbreeding estimates for individuals of each population are given in Figure 3 and Figure S1, respectively.

## 4 DISCUSSION

We examined the importance of genetic drift, including historical inbreeding and the contemporary mating system, in determining the genetic diversity and structure in populations of the freshwater snail, *L. stagnalis*, inhabiting ponds. We found a strong population genetic structure among five geographically closely located populations. The genetic differentiation of populations showed no obvious spatial pattern; even nearby populations (a few km apart) exhibited large genetic differences in allele frequencies. Patterns in genetic variation suggested genetic drift and historical inbreeding (e.g., due to founder events and/or small population size) in the examined populations observed as (1) large variation in *N*_e_, (2) a positive correlation between *N*_e_ and genetic variation, (3) the absence of rare alleles indicated by positive Tajima's *D* estimates, and (4) a negative correlation between Tajima's *D* estimates and genetic variation of the populations. In contrast, we did not find evidence for a strong effect of the contemporary mating system on the genetic diversity and structure because of (1) low selfing rates and low relatedness estimates in all populations and (2) the lack of a correlation between selfing rates and genetic variation of the populations. Our analyses suggest that populations of *L. stagnalis* inhabiting ponds likely exhibit extinction/(re)colonization dynamics and that their populations are shaped by founder events with limited influence of their contemporary mating system.

Strong population genetic structure is frequently reported in previous empirical studies on species with high rates of selfing or biparental inbreeding (Duminil et al., 2007; Jarne, 1995; Jullien et al., 2019). The observed large variation in genetic diversity and strong differentiation among populations align with earlier studies examining *L. stagnalis* populations using microsatellite markers in Switzerland, Finland, and New Zealand (Kopp et al., 2012; Puurtinen et al., 2004). Furthermore, covariation between the strength of signals related to genetic drift (e.g., *N*_e_ estimates and Tajima's *D* values) and genetic variation estimates of populations suggests that larger populations are less likely to experience local extinctions than small populations (Hill, 1981; Laurie‐Ahlberg & Weir, 1979). On the other hand, we did not find evidence for contemporary self‐fertilization driving the differences in diversity among the studied populations because selfing rates were low and did not covary with the genetic diversity estimates of the populations. For example, the most diverse populations (Hönggerberg and Warth‐Weiningen) had similar selfing rates to the least diverse ones (Kyburg and Witikon). However, self‐fertilization during the founding of populations might have strengthened genetic differentiation among populations (Ingvarsson, 1998, 2002). This is because, in self‐compatible hermaphrodites, self‐fertilization may further reduce the *N*_e_ by decreasing the number of independent gametes (Burgess et al., 2019; Jarne, 1995).

Although we found signs of nonrandom mating at the population level, our analysis did not reveal evidence for strong biparental inbreeding. This is because of low and similar mean values of pairwise relatedness estimates among populations. However, occasional self‐fertilization most probably leads to deviations from Hardy–Weinberg equilibrium in all populations, observed as excess homozygosity in this study. An alternative explanation for excess homozygosity could be within‐population structure (i.e., Wahlund effect). This effect arises when the sampled population is composed of subpopulations with varying allele frequencies. However, because there is no visible substructure within populations based on pairwise relatedness distributions and the PCA results, the Wahlund effect is unlikely to influence our study populations. Previous studies on *L. stagnalis* populations mostly found limited inbreeding depression (Coutellec & Lagadic, 2006; Koene et al., 2008; Puurtinen et al., 2007), but see Leicht et al. (2019) and Seppälä and Langeloh (2016), suggesting that recessive deleterious mutations may have been purged by population bottlenecks and founder events. However, these predictions have not previously been validated by genome‐wide markers. We suggest that further research employing genome‐wide analyses investigate the interplay between inbreeding, genetic diversity, demographic history, and life‐history traits in small populations experiencing extinction‐recolonization dynamics.

However, because we only examined five populations, the lack of significant correlations between mating system estimates and genetic diversity could be due to low statistical power. Furthermore, there are potential weaknesses in the SNP data when estimating genetic diversity and Tajima's *D*. This is because of systematic biases such as allelic dropouts, which might lead to decreased diversity and increased population differentiation estimates (Andrews et al., 2016; Arnold et al., 2013). We minimized these issues using a very high sequencing depth (mean site depth: 253), low mean missing data (1%), and the same sequencing and filtering criteria for all populations (Arnold et al., 2013). It is also important to note that rare alleles can introduce some bias in LD estimates (Do et al., 2014; Wang et al., 2016). However, we expect there to be little bias in LDN_e_ estimates due to rare alleles. This is because (1) our populations already exhibit a general deficit of rare alleles even before applying any minor allele filtering; (2) we used the same filtering thresholds for all of our populations; and (3) we tested the effect of minor allele filtering on LDN_e_ estimates and there was no difference in the results.

Limited gene flow in *L. stagnalis* populations, potentially caused by extinction‐recolonization cycles, likely explains the observed deficit of rare alleles (Wakeley, 2004). Self‐fertilization during founder events and/or occasional biparental inbreeding and self‐fertilization might further accentuate this effect (Bousset et al., 2004; Kopp et al., 2012; Puurtinen et al., 2004). While genetic drift may lead to lower evolutionary potential to respond to environmental change (Hoffmann et al., 2017), it can also transiently increase adaptive potential by epistatic or dominance variance to additive genetic variance by fixing common alleles in populations (Barton & Turelli, 2004). However, in the long term, repeated founder events and inbreeding are expected to slow down responses to directional selection due to decreased genetic variation. This aligns with findings from other freshwater snails (Escobar et al., 2011; Jarne et al., 2021; Lamy et al., 2012) and even organisms with larger populations (Sin et al., 2021), suggesting that demographic history can be a major driver of genetic diversity and inbreeding across species.

Understanding the timescales of genetic signals related to mating systems (recent) and genetic drift (historical) is crucial for interpreting their ecological significance. Some metrics, like identity disequilibrium, relatedness, and *F*_IS_, reflect recent mating patterns, typically corresponding to the last few generations. Conversely, others, such as π or Tajima's *D*, provide insights into longer‐term trends as they capture genetic variation accumulated over longer timescales, potentially including past inbreeding events. For example, selfing estimates derived from identity disequilibrium (ID) offer a snapshot of contemporary breeding patterns. This is because ID focuses on the probability of encountering identical genotypes at two loci due to recent ancestry (David et al., 2007; Ritland et al., 1990). On the other hand, linkage disequilibrium decays at longer timescales through recombination; therefore, linkage disequilibrium provides information about historical processes affecting population size, including bottlenecks (Hill & Robertson, 1968; Slatkin, 2008). Tajima's *D* estimates also potentially reflect more historical events, which lead to differences in the frequency of the rare alleles (Tajima, 1989). Thus, while our study found no strong evidence for recent selfing or biparental inbreeding, we cannot definitely rule out their historical influence on genetic diversity. Demographic simulations that incorporate various mating system parameters and extinction/recolonization events would be very useful for gaining a more comprehensive understanding of the demographic processes shaping these populations and also potential biases arising from different timescales of genetic signals.

In conclusion, our results suggest that founder effects and genetic drift play a larger role in defining the population genetic structure of *L. stagnalis* in the studied region than the contemporary mating system. Previous studies analyzing the role of genetic drift and the mating system using genomic techniques compared different taxa (Diaz‐Martin et al., 2023; Latron et al., 2020) or only one population (Sin et al., 2021). Our results illustrate how the investigation of population genetic diversity and structure in small populations characterized by a mixed‐mating system benefits from genome‐wide multilocus analyses. The differences in genetic drift and diversity among populations that we report might reflect the degree to which genetic drift and/or founder events, including historical inbreeding, erode the adaptive potential of small populations (Lynch et al., 1995). However, if those populations undergo extinction/(re)colonization dynamics frequently, genetic diversity and adaptive potential would be largely determined by the source populations. Thus, investigating small ponds and more permanent habitats to identify potential source and sink populations would be necessary to understand the relevant spatial units for biodiversity conservation.

## AUTHOR CONTRIBUTIONS

**Cansu Çetin:** Formal analysis (lead); investigation (equal); methodology (equal); writing – original draft (lead); writing – review and editing (equal). **Jukka Jokela:** Resources (equal); supervision (equal); validation (equal); writing – original draft (supporting); writing – review and editing (equal). **Philine G. D. Feulner:** Formal analysis (supporting); methodology (equal); validation (equal); writing – review and editing (supporting). **Tamara Schlegel:** Methodology (supporting); writing – review and editing (supporting). **Nadine Tardent:** Methodology (supporting); writing – review and editing (supporting). **Otto Seppälä:** Conceptualization (lead); funding acquisition (lead); investigation (equal); methodology (lead); project administration (equal); resources (equal); supervision (equal); validation (equal); writing – review and editing (supporting).

## FUNDING INFORMATION

The study was funded by the ETH research commission (grant ETH‐20 17‐1) and SNF grant (31003A 169531) to OS.

## CONFLICT OF INTEREST STATEMENT

The authors declare no conflicts of interest.

### OPEN RESEARCH BADGES

This article has earned an Open Data badge for making publicly available the digitally‐shareable data necessary to reproduce the reported results.

## Supporting information


Data S1.

## Data Availability

Raw genetic data (fastq files for all 92 individuals) are deposited on short read archive, SRA under accession numbers SAMN36306817‐SAMN36306908. Genotypes (vcf format) are available at the dryad repository https://datadryad.org/stash/share/Oflxul7HzO0pe2yJ3il1o5GuvIEzGVhLr9HwlMBNx5g.
