# Supplementary material for: Population genetic structure in a self‐compatible hermaphroditic snail is driven by drift independently of its contemporary mating system
Source: Ecol Evol. 2024 Aug 13;14(8):e70162. doi: 10.1002/ece3.70162 (PMC11319733; doi:10.1002/ece3.70162)
Supplement: Supplementary file 1 — Data S1. [file ECE3-14-e70162-s001.docx]

**SUPPLEMENTARY FIGURES AND TABLES**

**Text S1. CTAB extraction and magnetic bead clean up steps for *Lymnaea stagnalis***

CTAB protocol was modified from Doyle and Doyle 1987).

1. Take snails out of tube, dab dry and push out remaining ethanol
2. Grind samples with pestles and some quartz in 50 µl CTAB buffer
3. Top up CTAB to 300 µl
4. Add 15 µl of 2mg/ml Proteinase K
5. Put on thermoshaker at 55 °C overnight at 450 rpm
6. Add 150 µl of TE_low_
7. Add 5 µl of RNAse A 20mg/ml
8. Mix overend several times, spin down
9. Incubate for 30 min at 37 °C
10. Add 600 µl of chloroform:IAA (24:1)
11. Mix overend several times until a milky emulsion has formed
12. Spin for 10 min 15000 x *g* (=rcf)
13. Take up to 450 µl of supernatant to new tube (usually you only get around 300 because of all the tissue)
14. Add another 600 µl of chloroform:IAA (24:1)
15. Mix overend several times (approx. 20 times) [it is much harder now to get an emulsion, just make sure that the two layers mix enough times for remaining proteins etc. to go into the organic phase)
16. Spin for 10 min 15000 x *g* (=rcf)
17. Take supernatant to new 2mL tube
18. Add 45 µl Sodium Acetate (3M)
19. Add 900 µl ice-cold 100% isopropanol
20. Mix overend several times
21. Precipitate 1h in -20°C freezer
22. Spin 25 min at 4°C
23. Wash
    1. Check pellet and remove supernatant

*x* 2

- 1. Add 900 µl ice-cold 70% EtOH
  2. Spin 10min

1. Check pellet and remove supernatant [pellet will most likely be dark in color)
2. Spin down quickly, remove remaining EtOH with 10 µl pipette.
3. Dry pellets on bench for 20-30 minutes (check periodically, they should not overdry!)
4. Add 100 µl TE_low_
5. Put on thermoshaker at 37 – 40 °C, wait until pellet dissolves, flick occasionally
6. Spin down
7. Proceed with magnetic bead clean up.
8. Take CTAB extracted DNA in 2 ml tube and add one volume of binding buffer and 1:18 (v:v) of magnetic beads previously prepared (vortex the beads solution for 20 seconds before use to ensure that the beads are completely resuspended).
9. Mix by inverting the tube 20 times
10. Incubate with a gentle agitation (with a rotator or a rocker platform) for 10 minutes at room temperature.
11. Spin down the tube for 1 second.
12. Place the tube in a magnetic rack for 3 minutes (until the solution becomes clear). The actual time required to collect beads may vary according to samples.
13. Remove the supernatant without disturbing the beads pellet.
14. Add 1 ml of wash solution, remove the tube from the magnetic rack and mix by inverting the tube 20 times.
15. Spin down the tube for 1 second.
16. Place the tube in the magnetic rack and wait for 30 seconds (until the solution becomes clear).
17. Remove the supernatant without disturbing the beads pellet.
18. Repeat steps 7 to 10
19. Spin down the tube for 1 second and place the tube on the magnetic rack to remove the remaining washing solution.
20. Let the beads air-dry for 1 minute with the cap open. Do not let the beads dry more than 1 minute as this will significantly decrease elution efficiency.
21. Add 80 μl of the elution buffer TE_low_ preheated to 50°C.

| **** |
| --- |

**Fig S1.** Inbreeding coefficients (*f*) from the probability-based estimator, TrioML (Wang 2007) performed on each of the five populations separately, thus using within-population allele frequencies in corresponding populations. The red lines indicate the mean values for each population.

**Table S1.** SNP filtering protocol adapted from the dDocent pipeline (Puritz et al. 2014).

| **# of retained SNPs after filtering** | **# Ind** | **tools** | **commands** | **Filter explanation** |
| --- | --- | --- | --- | --- |
| 314 016 | 93 |  |  |  |
| 19 445 | 93 | vcftools | *--max-missing 0.5 --mac 3 --minQ 30* | Remove sites with high missingness |
| 19 445 | 93 | vcftools | *--minDP 3* | Minimum read depth |
| 19 445 | 92 | vcftools | *--remove-indv 003.WT* | Remove bad quality individual |
| 16 515 | 92 | vcftools | *--min-meanDP 16* | Based on the depth histogram, I decide to exclude sites with mean depth < 15 |
| 15 706 | 92 | vcftools | *--max-missing 0.50* | Missingness filter |
| 10 622 | 92 | vcftools | *--exclude-positions badloci* | Excluding all loci with >20% missing data in at least 1 population |
| 10 264 | 92 | vcffilter | *-s -f "AB > 0.25 & AB < 0.75 \| AB < 0.01* | Allele balance filter |
| 10 168 | 92 | vcffilter | *-f "SAF / SAR > 100 & SRF / SRR > 100 \| SAR / SAF > 100 & SRR / SRF > 100" -s* | Filtering out sites that have reads from both strands |
| 7 097 | 92 | vcffilter | *-f "MQM / MQMR > 0.9 & MQM / MQMR < 1.05* | Filter for mapping quality between reference and alternate alleles |
| 6 869 | 92 | vcffilter | *-f "QUAL / DP > 0.25"* | Filter for ratio of quality score to depth |
| 5 583 | 92 | vcftools | *--exclude-positions lowQDloci* | Filter loci with extremely high coverage |
| 5 471 | 92 | vcftools | *--max-meanDP 550 --min-meanDP 10* | Based on the depth histogram, I decide to exclude sites with mean depth > 550 and < 10 as it can indicate paralogs, multicopy loci or genotyping errors |
| 4 350 | 92 | vcftools | *--min-alleles 2 --max-alleles 2 --remove-indels* | Keep only biallelic sites and remove indels |
| 4 345 | 92 | vcftools | *--max-missing 0.80* | More stringent missingness filter |
| 4 232 | 92 | vcftools | *--exclude-bed DenseLoci4345_2.bed* | removing loci w more than 5 SNPs |
| 4 054 | 92 | vcftools | *--mac 5* | minor allele filtering, “non-thinned dataset” |
| 2 263 | 92 | vcftools | *--thin 170* | Keeping one SNP per locus (2x85 bp), “thinned dataset |

**Table S2.** Pearson correlations among among nucleotide diversity (*π*) and environmental characteristics of ponds (elevation and area of the ponds) and the most relevant measures for drift (*N*_e_ and Tajima’s *D*) and mating type (s(g_2_)). Significant estimates (*p* < 0.05) are shown in bold.

|  | ***π*** | ***N*_e_** | **Tajima’s D** |
| --- | --- | --- | --- |
| **Area (m^2^)** | -0.44 (0.56) |  |  |
| **Elevation (m)** | -0.86 (0.14) |  |  |
| ***N*_e_** | **0.98 (0.02)** |  |  |
| **Tajima’s D** | **-0.95 (0.05)** | -0.88 (0.12) |  |
| **s(g_2_)** | -0.72 (0.28) | -0.56 (0.44) | 0.88 (0.12) |

Abbreviations used: Nucleotide diversity (*π)*; effective populations size (*N_e_*); selfing rate, s(g_2_), calculated using identity disequilibrium.

**Table S3.** Estimates for Tajima’s *D* based on the non-thinned data without minor allele filtering (Total SNPs= 4232) are shown.

| **Population** | **Tajima’s *D*** |
| --- | --- |
| **Eschenberg** | 0.98 |
| **Hönggerberg** | 0.60 |
| **Warth-Weiningen** | 0.78 |
| **Kyburg** | 0.94 |
| **Witikon** | 1.19 |
